# Mitochondrial TrxR2 regulates metabolism and protects from metabolic disease through enhanced TCA and ETC function

**DOI:** 10.1038/s42003-022-03405-w

**Published:** 2022-05-16

**Authors:** E. Sandra Chocron, Kennedy Mdaki, Nisi Jiang, Jodie Cropper, Andrew M. Pickering

**Affiliations:** 1 Barshop Institute for Longevity and Aging Studies, University of Texas Health San Antonio, San Antonio, TX USA; 2 Department of Radiation Oncology, Long School of Medicine, University of Texas Health San Antonio, San Antonio, TX USA; 3 Center for Neurodegeneration and Experimental Therapeutics, Department of Neurology, University of Alabama at Birmingham, Birmingham, AL USA

**Keywords:** Energy metabolism, Cell biology

## Abstract

Mitochondrial dysfunction is a key driver of diabetes and other metabolic diseases. Mitochondrial redox state is highly impactful to metabolic function but the mechanism driving this is unclear. We generated a transgenic mouse which overexpressed the redox enzyme Thioredoxin Reductase 2 (TrxR2), the rate limiting enzyme in the mitochondrial thioredoxin system. We found augmentation of TrxR2 to enhance metabolism in mice under a normal diet and to increase resistance to high-fat diet induced metabolic dysfunction by both increasing glucose tolerance and decreasing fat deposition. We show this to be caused by increased mitochondrial function which is driven at least in part by enhancements to the tricarboxylic acid cycle and electron transport chain function. Our findings demonstrate a role for TrxR2 and mitochondrial thioredoxin as metabolic regulators and show a critical role for redox enzymes in controlling functionality of key mitochondrial metabolic systems.

## Abstract (web summary)

TrxR2 overexpression in mice enhances metabolism and prevents high-fat diet induced metabolic deficits in mice through alteration in function of several enzymes in the tricarboxylic acid cycle and complexes of the electron transport chain.

## Introduction

Metabolic syndrome is a prevalent public health issue affecting one in three adults in the United States^1^. Mitochondrial dysfunction is a prominent driver of metabolic deficits and has also been linked with Alzheimer’s disease, Parkinson’s disease, Huntington’s disease, Amyotrophic Lateral Sclerosis, Friedreich’s ataxia, cardiovascular disease, atherosclerosis, and diabetes^2^. Impaired mitochondrial function has been linked with metabolic deficits, elevated serum glucose levels, diminished glucose tolerance, and increased insulin resistance, all of which are driving factors for diabetes progression^3^. Declines in mitochondrial function and metabolic deficits are well-cataloged aspects of aging and are likely a key driver in age-related increases in diabetes incidence rates^4^. The connection between mitochondrial function and diabetes is particularly apparent in studies of mitochondrial redox enzymes. Transgenic expression of mitochondrial-targeted catalase (mCAT) prevented age-associated declines in mitochondrial function and decreased insulin resistance in muscle^5^. Overexpression of the mitochondrial peroxidase PRX3 was shown to decrease fasting plasma glucose levels and protect against high-fat diet-induced glucose intolerance^6^. However, the mechanism through which increased mitochondrial redox scavenging regulates metabolic function remains unclear.

Thioredoxin is a redox protein whose primary function is the scavenging of oxidants and free radicals through reduction of protein disulfide bonds into thiolate anion^7^. There are two major forms of thioredoxin in the cell, thioredoxin-1 which is cytosolic and thioredoxin-2 which is mitochondrial^8^. Thioredoxin-2 represents the major redox scavenging system in mitochondria^9^ with multiple functions in this organelle. It reduces oxidized proteins allowing the repair of oxidative damage in mitochondria^7^. It plays a key role in the peroxiredoxin (PRX) system. PRX is oxidized as part of its role in the reduction of hydrogen peroxide into water and is another substrate for thioredoxin-2 allowing restoration of its function^10^. Thioredoxin-2 also plays a key role in the regulation of cell death by repressing ASK-1-regulated cell death apoptotic signaling pathways^11^. Thioredoxin-2 acts as a redox sensor and becomes oxidized under oxidative stress conditions de-repressing ASK-1 and triggering the apoptotic pathway^11^.

The enzyme thioredoxin reductase 2 (TrxR2) is critical for the continued function of thioredoxin-2. TrxR2 reduces oxidized thioredoxin-2 in a reaction involving the conversion of NAD(P)H to NAD(P)^+^. This is a critical rate-limiting step in the function of Thioredoxin-2^12^. TrxR2 levels have been reported to decline in rat skeletal and cardiac muscle over the course of age, driving age-related declines in mitochondrial redox capacity^13^. We have previously reported that augmentation of TrxR2 function can extend lifespan in *Drosophila melanogaster* and diminish age-related declines in oxygen consumption^14^. All these previous findings suggested that TrxR2 may play an important role in the regulation of mitochondrial metabolic function and potentially protect against metabolic disease.

In this study, we created a TrxR2 overexpression mouse. We found TrxR2 overexpression to enhance metabolism and prevent high-fat diet-induced metabolic deficits in mice. We found TrxR2 overexpression induced metabolic improvement through alteration in the function of several enzymes in the Tricarboxylic acid (TCA) cycle and complexes of the electron transport chain. Our findings demonstrate a role for the thioredoxin system as a metabolic regulator impacting key systems of mitochondrial function.

## Results

### Thioredoxin reductase 2 overexpression mouse model

**Fig. 1:**
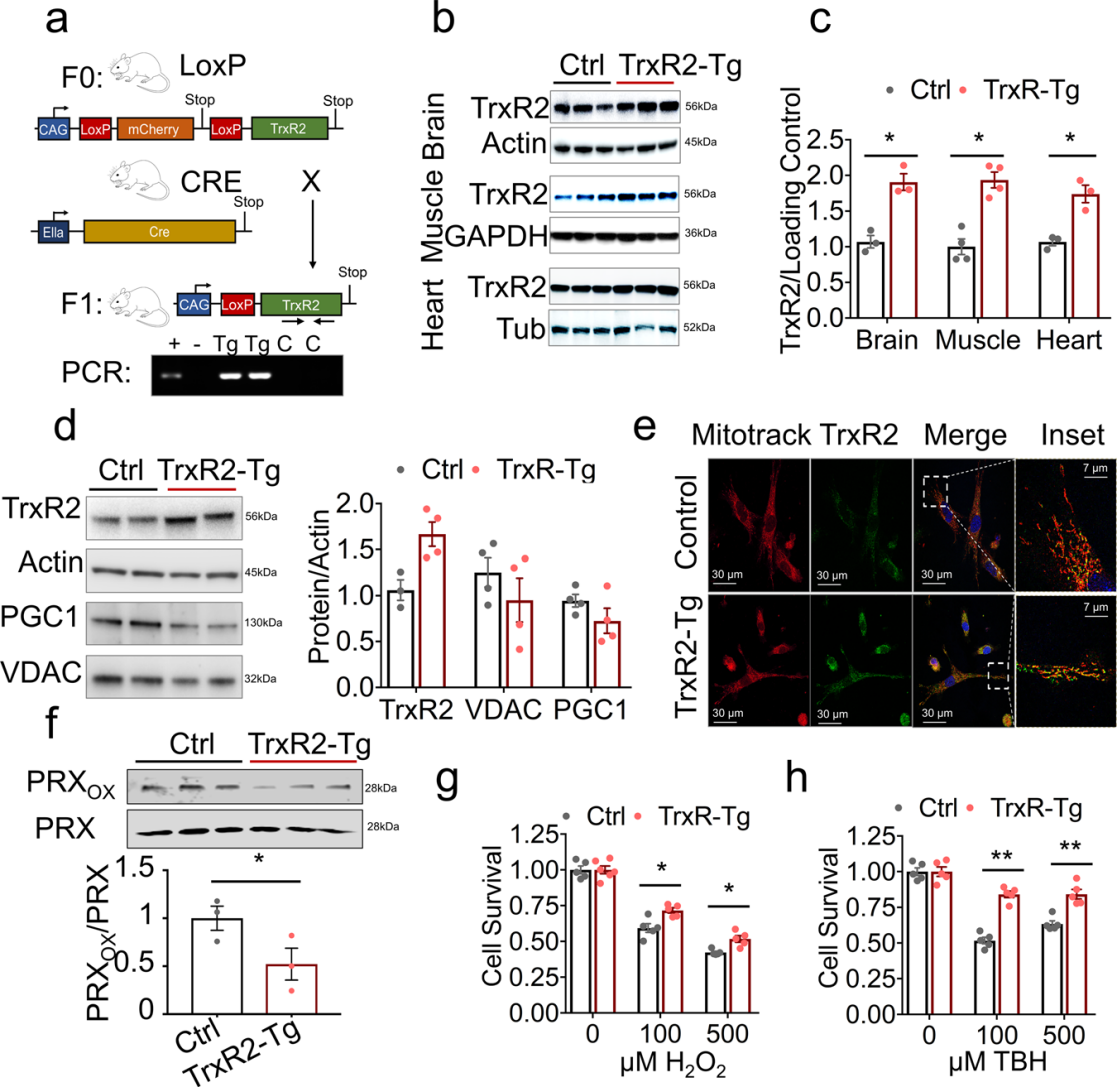
Characterization of TrxR2-Tg mouse. **a** Breeding schematic of CAG-LoxP-STOP-LoxP-TrxR2 transgenic mice that were bred with EIIA-CRE mice producing CAG-LoxP-TrxR2 mice (referred to as TrxR2-Tg). **b**, **c** TrxR2 is elevated in TrxR2-Tg mice. Immunoblots and quantifications of TrxR2 in the brain, muscle, and heart tissues from 10-month-old TrxR2-Tg mice and littermate controls (*n* = 3). **d** TrxR2 overexpression does not alter mitochondrial abundance. Immunoblot of TrxR2, VDAC, and PGC-1 in MEFs derived from TrxR2-Tg mice (*n* = 3). **e** TrxR2 overexpression is localized in mitochondria. Immunofluoresence of TrxR2-Tg and non-transgenic MEFs stained with mitotracker, representative images. **f** Western blot of oxidized and total levels PRX protein in TrxR2-Tg versus control liver mitochondria isolates (*n* = 3). **g** TrxR2 overexpression increases oxidative stress resistance. H_2_O_2_ cell survival assay of TrxR2-Tg and Control MEFS (*n* = 3). **h** *Tert*-butyl hydroperoxide cell survival assay of TrxR2-TG MEFs and non-transgenic MEFS (*n* = 3). Values are Mean ± SEM. *p < 0.05, **p < 0.01.

We designed a TrxR2 overexpression mouse through the insertion of a transgene containing a single copy of mouse TrxR2 cDNA preceded by a ubiquitous CAG promoter and two LoxP sequences sandwiching a premature stop site. Under these conditions, the TrxR2 gene is not translated and therefore not overexpressed. We then crossed this mouse with a ubiquitous homozygous Cre recombinant overexpressor (EIIa-CRE) which led to the deletion of the premature stop site generating a ‘CAG-TrxR2’ overexpression mouse (Fig. 1a). The mice produced with this transgene (TrxR2-Tg) were viable, fertile, and physiologically normal with no observed deficits. We validated overexpression through western blot and demonstrated a 75–100% increase in TrxR2 expression levels in the brain, muscle, and heart (Fig. 1b, c). We also observed similar increases in liver and lung tissues (Supplementary Fig. 1a, b).

### TrxR2 overexpression is mitochondrial localized, reduced mitochondrial redox state, and increased oxidative stress resistance

To assess cellular localization and functionality of TrxR2 we derived Mouse Embryonic Fibroblasts (MEFs) from TrxR2-Tg mice. TrxR2-Tg MEFs showed a 50% increase in TrxR2 protein levels. We did not detect any significant changes in mitochondrial markers PGC-1α or VDAC levels (Fig. 1d) or in mtDNA copy number (Supplementary Fig. 1c). This suggested that both mitochondrial biogenesis and mitochondrial mass remained unchanged in MEFs or tissues derived from TrxR2 transgenic mice. We confirmed TrxR2 mitochondrial co-localization with immunofluorescence using mitotracker. As confirmed by western blot we saw a higher expression of TrxR2 in TrxR2-Tg MEFs (green, Fig. 1e) and demonstrated TrxR2 protein overexpression was predominantly co-localized with mitochondria (Merge, Fig. 1e). In addition, we demonstrated that liver mitochondrial isolates derived from TrxR2-Tg mice exhibited lower oxidized PRX3 levels suggesting higher functionality of thioredoxin-2 (Fig. 1f). We also found TrxR2-Tg MEFs were more resistant to H_2_O_2_ treatment than MEFs derived from littermate controls and displayed higher protection against the mitochondrial-specific oxidative stressor Tert-butyl hydroperoxide^15^ (TBH) (Fig. 1g, h). These findings support TrxR2 overexpression being mitochondrial-specific TrxR2 overexpression and increasing TrxR2 functionality.

### TrxR2-Tg mice show improved glucose tolerance

**Fig. 2:**
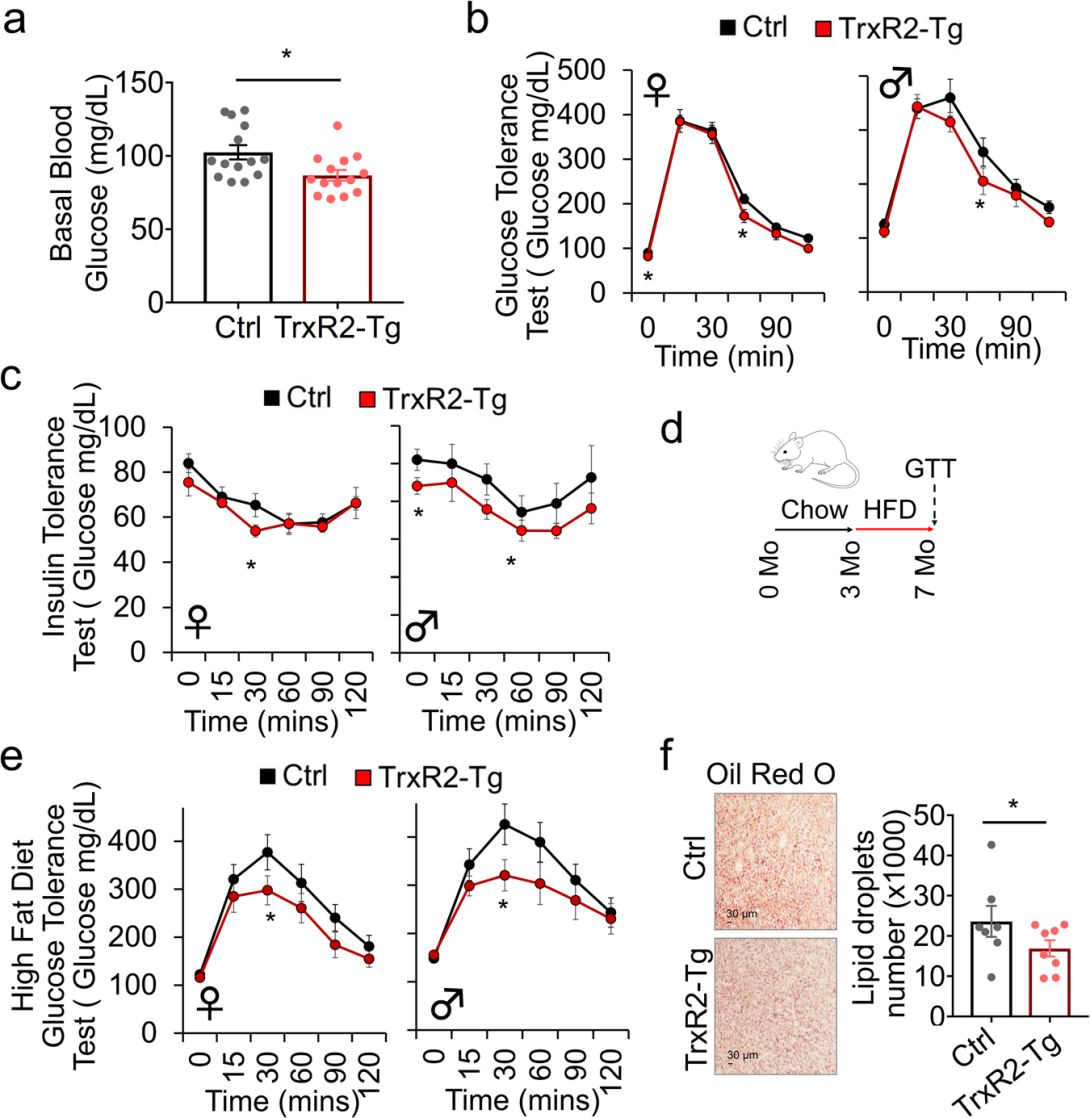
Glucose metabolism and tolerance is improved in TrxR2-Tg mice maintained on standard chow and high-fat diet. **a** TrxR2-Tg mice have lower basal blood glucose, 7 months old mice (ANOVA with Tukey’s test, *n* = 7–9). **b** Chow-fed 8-month-old TrxR2-TG mice show improved glucose tolerance compared to littermate controls (*n* = 7–9) separate tests were done for female and male groups. **c** Chow-fed 8-month-old TrxR2-TG mice have higher insulin sensitivity than littermate controls separate tests were done for female and male groups (*n* = 8–9). **d** Schematic of high-fat diet feeding regime used in panels **e**, **f**. **e** High-fat diet-fed 8 months old TrxR2-Tg mice show improved glucose tolerance compared to littermate controls separate tests were done for female and male groups (*n* = 8–10). **f** High-fat diet-fed 8 months old, males and females TrxR2-Tg mice have decreased liver fat compared to littermate controls. Oil Red O stain of liver slices, *n* = 8. Values are Mean ± SEM. *p < 0.05, **p < 0.01.

We hypothesized that overexpression of TrxR2 may influence metabolism. To assess this, we measured fasting blood glucose levels in TrxR2-Tg mice. Interestingly, we found TrxR2-Tg mice to have lower basal blood glucose levels than littermate controls (Fig. 2a). We hypothesized that this may be driven by the enhanced capacity to metabolize glucose. To test this, we monitored blood glucose clearance using glucose tolerance tests (1.5 g/kg glucose bolus injection). We were interested to find that both male and female TrxR2-Tg mice could metabolize glucose slightly faster than littermate controls showing improved glucose tolerance (Fig. 2b). In addition, both male and female TrxR2-Tg mice displayed slightly increased insulin sensitivity measured through insulin tolerance tests (0.8 U/Kg insulin injection) (Fig. 2c). These findings suggest TrxR2 overexpression to improve metabolic function in adult mice. These improvements made us hypothesize that TrxR2 may be protective against metabolic disease. To investigate this, we placed mice on a high-fat diet for 4 months (Fig. 2d). We found both male and female high-fat diet-fed TrxR2-TG mice displayed significantly improved glucose tolerance relative to high-fat diet-fed littermate controls (Fig. 2e). Surprisingly though no difference in insulin sensitivity was observed (Supplementary Fig. 2).

We found mouse weight to be lower in male but not female TrxR2-Tg mice maintained on a normal diet when compared to littermate controls (Supplementary Fig. 3a, b). However, we did not observe any significant changes in weight in mice maintained on a high-fat diet (Supplementary Fig. 3c,d). Despite the lack of change in weight we observed decreased liver lipid content suggesting reduced liver steatosis in high-fat diet maintained TrxR2-Tg mice compared to littermate controls (Fig. 2f). Our findings suggest TrxR2 overexpression to increase basal glucose metabolism and improve glucose tolerance in both adult mice and mice fed with a high-fat diet. In addition, while we observed some improvements in insulin sensitivity under a normal diet, high-fat diet experiments suggested this response to be at least partly independent of insulin sensitivity.

### TrxR2-Tg male mice exhibit improved whole-body metabolism

**Fig. 3:**
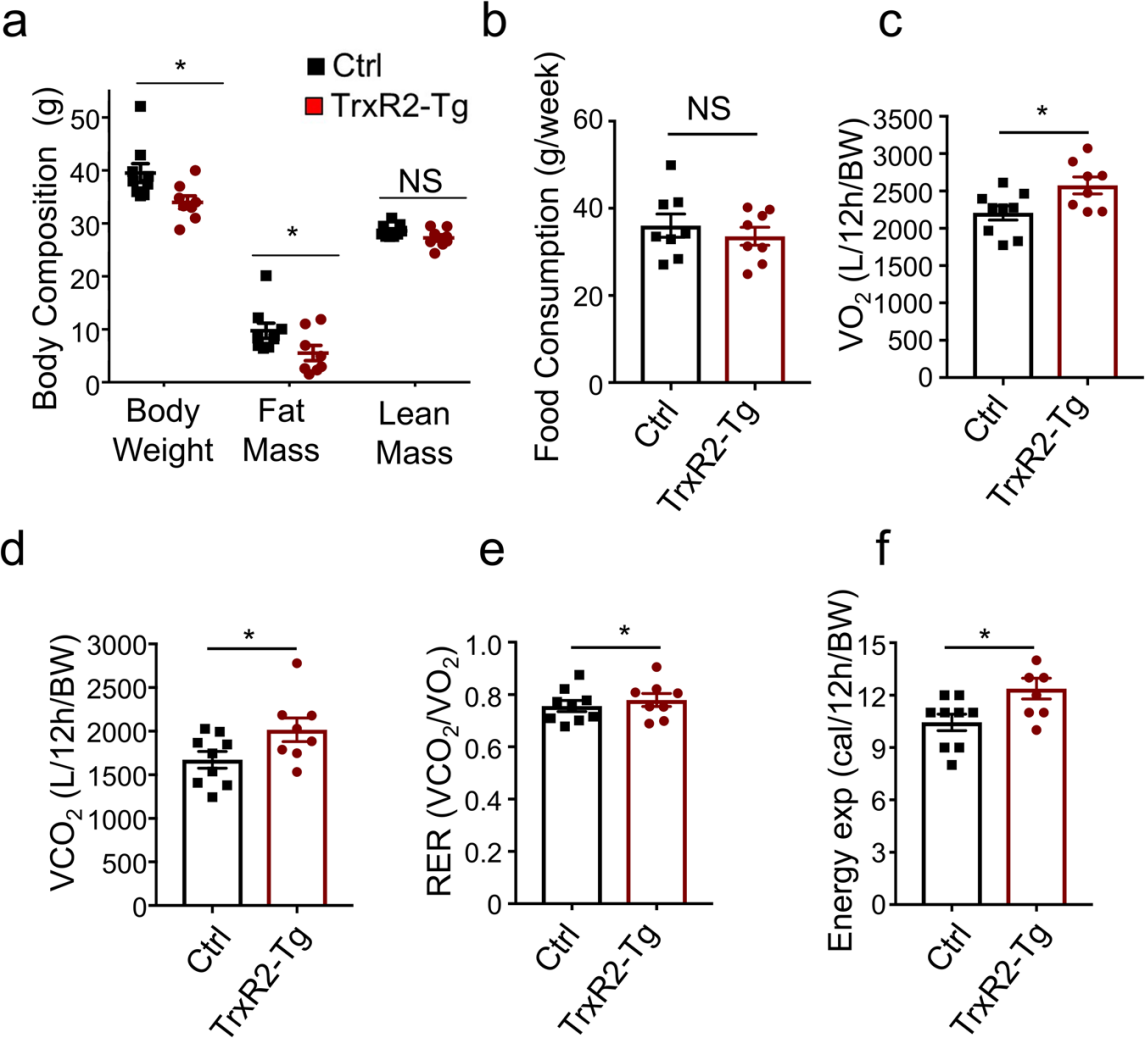
TrxR2 overexpression increases light cycle whole-body metabolism in the normal diet-fed male mice. **a** TrxR2-TG male mice are leaner with lower weight and fat mass relative to littermate controls as measured by qMRI (see methods, *n* = 8–9). **b** Food consumption is unaltered. Food intake was recorded for 7 days, (*n* = 8–9). **c**–**f** Indirect calorimetry was performed in normal-fed male mice. Parameters for each mouse were obtained and normalized by body weight. **c** Daytime O_2_ consumption (VO_2_). **d** CO_2_ production (VCO_2_). **e** RER (CO_2_/O_2_), and **f** energy expenditure (Heat). All experiments utilized 8 months old normal-fed male mice, *n* = 8–9. Values are Mean ± SEM. *p < 0.05.

To gain a greater understanding of the changes produced by TrxR2 overexpression we examined the impact of TrxR2 overexpression on whole-body composition and metabolism. In normal-fed male mice, we observed a significant decline in body weight as well as changes in body composition (Fig. 3a). We were interested to find a significant decline in fat mass in TrxR2-Tg mice, in contrast, we did not see any significant change in lean mass in these mice (Fig. 3a). These findings suggested that overexpression of TrxR2 increased metabolic processes and decreased fat mass. Importantly, we did not observe any changes in food consumption with TrxR2 overexpression suggesting the changes observed in weight were not due to decreased food intake (Fig. 3b). We monitored other metabolic parameters using indirect calorimetry in individualized cages. Importantly we observed that TrxR2-Tg mice show increased oxygen consumption, CO_2_ production, and energy expenditure (Heat) without changes in Respiratory Exchange Ratio (RER) during the light cycle (Figs. 3c–f). While no changes were observed during the dark cycle (Supplementary Fig. 4a–d). These results suggest that basal metabolism is increased with TrxR2 overexpression, however at night when mice are more active transgenic animals did not display a higher energy demand than littermate controls. Furthermore, we did not see any changes in RER suggesting there was not a metabolic switch occurring in the transgenic animals with no change in substrate preference between carbohydrates and fat. All these findings suggest that increasing TrxR2 expression increases basal metabolism in mice leading to decreased fat mass.

### TrxR2-Tg isolated liver mitochondria exhibit higher membrane potential and respiration

**Fig. 4:**
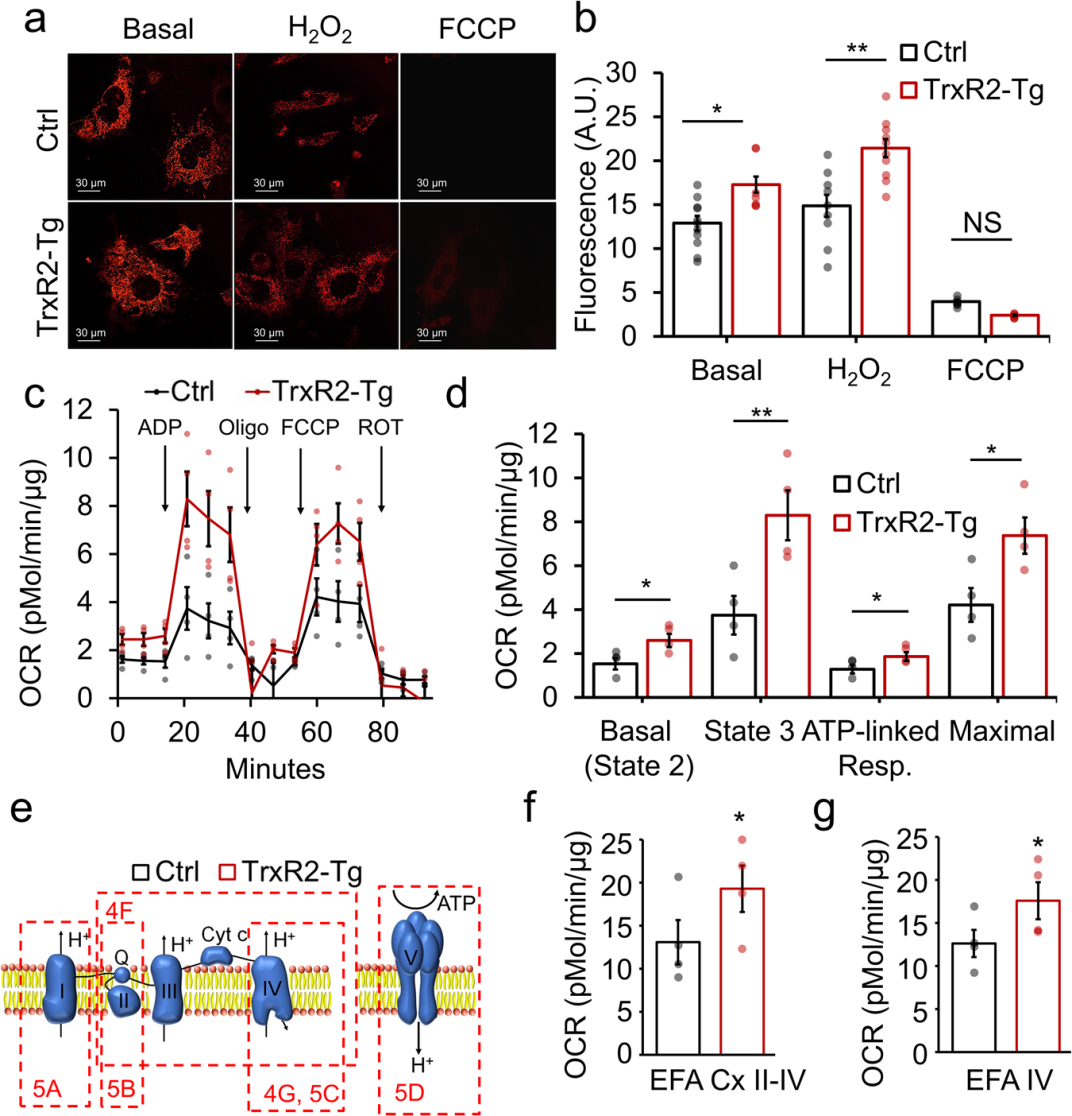
TrxR2-Tg MEFs display elevated mitochondrial membrane potential and electron transport chain function. **a** TrxR2-Tg MEFs live-cell confocal imaging with 10 nm TMRM. **b** TMRM fluorescence intensity was analyzed with ImageJ and TrxR2-Tg MEFs display higher mitochondrial membrane potential than non-transgenic controls (*n* = 3 with triplicates). **c** Coupling seahorse assay showing oxygen consumption rate (OCR) trace with injection sequence of mitochondria isolated from livers of TrxR2-TG mice and littermate controls (*n* = 4). **d** All calculated respiratory parameters are increased in TrxR2-Tg mitochondria relative to non-transgenic controls (*n* = 4 mice with 10 technical replicates). **e** Schematics of the ETC are used to depict the sequence of complexes measured for each figure panel. **f** Seahorse electron flow assay (EFA) assessing respiratory flow from complex II to complex IV (*n* = 4). **g** Seahorse electron flow assay (EFA) assessing Complex IV respiration. Values are Mean ± SEM. *p < 0.05.

We sought to understand the mechanism through which TrxR2 overexpression was enhancing metabolic function. We found TrxR2-Tg MEFs to display increased mitochondrial membrane potential (MMP), measured with tetramethylrhodamine methyl ester (TMRM) in live cells. We found TrxR2-Tg MEFs to show increased MMP both under basal and acute stress conditions with hydrogen peroxide (Fig. 4a, b). Interestingly hydrogen peroxide did not decrease the MMP probably due to the acute nature of the exposure (30 min). We found TMRM staining to be specific since it faded after exposure to 10 µM FCCP as expected (Fig. 4a, b). We hypothesized that the TrxR2-induced increase in membrane potential might be driven by increases in mitochondrial respiration. To test this, we performed seahorse coupling respiratory assays in isolated mitochondria obtained from TrxR2-Tg and control mice livers. TrxR2-Tg seahorse traces appeared higher than control mouse traces throughout the assay (Fig. 4c). We noted that TrxR2-Tg mitochondria displayed a significant increase in basal oxygen consumption rate in the presence of pyruvate and malate, State 3-ADP stimulated respiration, ATP-coupled respiration, and maximal respiration when compared to littermate controls (Fig. 4c, d).

### TrxR2 overexpression enhances the electron transport chain function

**Fig. 5:**
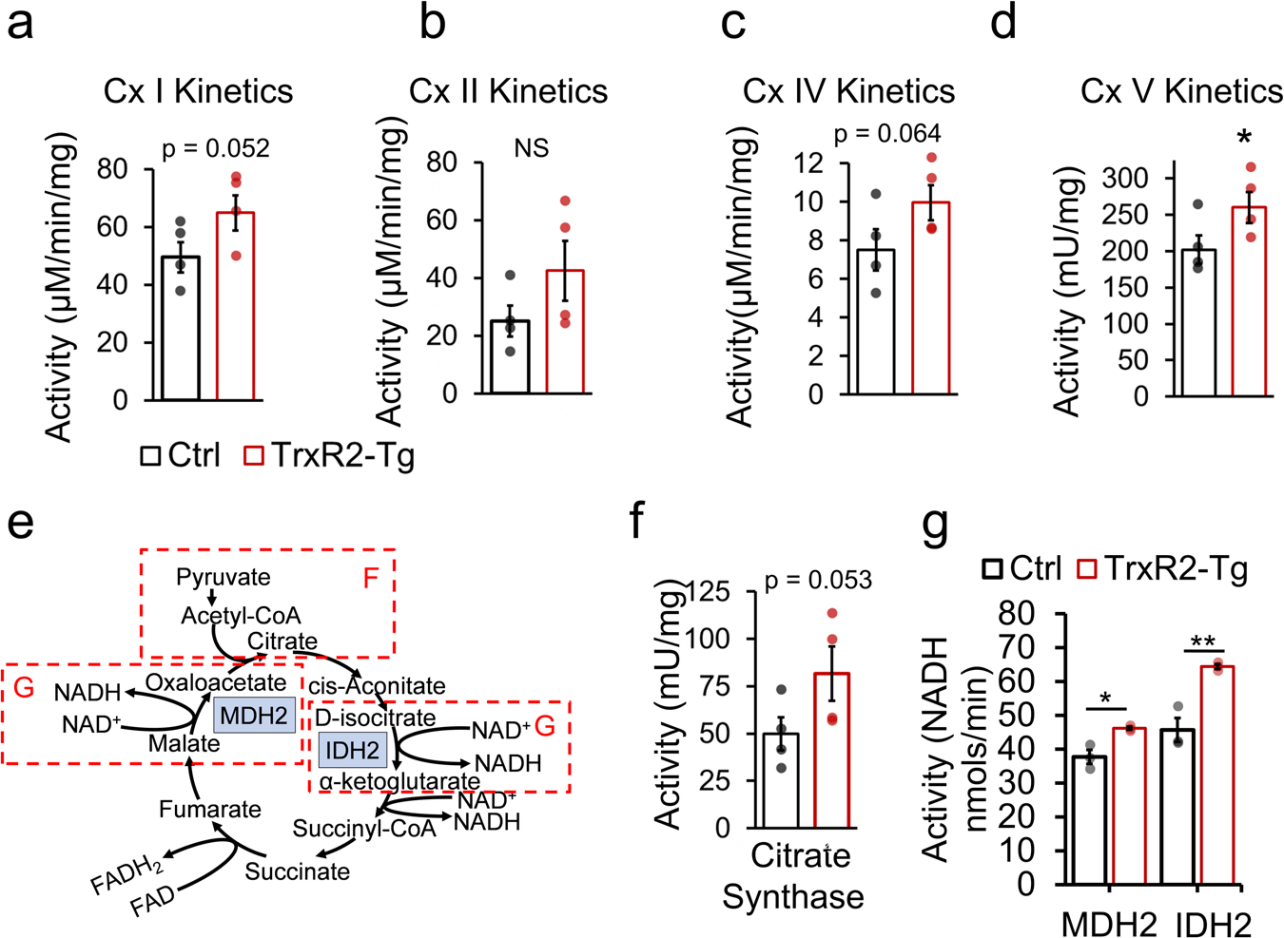
TrxR2-Tg liver isolated mitochondria display increased function through direct regulation of the tricarboxylic acid cycle and the electron transport chain (ETC). **a**–**d** Individual kinetic activities for Complex I (Cx I), complex II (Cx II), complex IV (Cx IV), and complex V-ATP Synthase (Cx V) respectively in TrxR2-Tg mitochondria compared to controls. **e** Schematics of the tricarboxylic acid cycle (TCA) indicating with dashed lines the section referred. **f** Citrate synthase activity kinetics was evaluated (*n* = 4). **g** The specific function of the TCA cycle enzymes MDH2 and IDH2 were increased in TrxR2-Tg mitochondria relative to non-transgenic controls, evaluated by MDH2 and IDH2 activity assays. (*n* = 3, assays run in triplicates). Values are Mean ± SEM. *p < 0.05, **p < 0.01.

To understand the mechanism through which TrxR2 overexpression was enhancing respiratory function we first examined impacts on the electron transport chain (ETC) by electron flow seahorse assays. This approach evaluates ETC respiratory function uncoupled from ATP production by ATP Synthase. We used the same set of isolated mitochondria from TrxR2-Tg and control livers as those used in Fig. 4c, d. We observed significantly higher respiratory values from complexes II through IV (Fig. 4e, f) after the addition of Rotenone and Succinate and complex IV alone after the addition of Ascorbate and TMPD in TrxR2-Tg mitochondria compared to controls (Fig. 4g) (all in the presence of dinitrophenol and pyruvate and malate). We sought to understand whether these increases in ETC complexes respiratory values could be due to higher function of individual complexes activities. We found complex I activity to be elevated (*p* = 0.052), measured through kinetic activity assays (Fig. 5a)*.* In addition, we observed significantly elevated complex V-ATP Synthase activity (Fig. 5d) with a trend for elevations in complex II and IV (Fig. 5b, c). Complex III was not evaluated. These results are in line with our seahorse electron flow assays results indicating that enhancing Thioredoxin-2 function targets multiple components of the respiratory chain.

### TrxR2 overexpression enhances the tricarboxylic acid chain function

We hypothesized that TrxR2 overexpression may also impact other mitochondrial metabolic components that could lead to increased substrate availability feeding the ETC possibly through the tricarboxylic acid (TCA) cycle (Fig. 5e). We investigated this through activity measures of multiple components of the TCA cycle. We found citrate synthase (the first enzyme in the TCA cycle) to display a trend for higher activity in TrxR2-Tg versus controls (Fig. 5f). To investigate whether there could be changes in TCA cycle function specific to NADH generation we investigated other components of the TCA cycle such as malate dehydrogenase (MDH2) which converts malate to oxalacetate and isocitrate dehydrogenase (IDH2) which converts d-isocitrate to α-ketoglutarate. These targets were chosen as both were previously described to be direct molecular targets of cytoplasmic Thioredoxin-1 which is highly homologous to Thioredoxin-2, the substrate of TrxR2^16^. We found the functionality of both MDH2 and IDH2 to be increased in isolated mitochondria from TrxR2-Tg livers (Fig. 5g). These changes in function did not appear to be due to increased protein levels that could be caused by different factors. TrxR2-Tg showed similar protein levels of IDH3α, MDH2, and ATP5α in mitochondria when compared to controls. This strongly suggests that Thioredoxin-2 reduces oxidized thiol groups in multiple protein targets in mitochondria impacting their functionality (Supplementary Fig. 5a, b). All these findings suggest TrxR2 to exert a synergistic function to increase mitochondrial respiration impacting directly several components of the electron transport chain and at the same time increasing substrate availability by the TCA cycle.

## Discussion

Our findings show a key role for the mitochondrial thioredoxin system Thioredoxin-2/TrxR2 as a protector against metabolic dysfunction. Thioredoxin-2 acts upstream of the peroxiredoxin system thus our results are consistent with previous findings of enhanced metabolism by PRX3 overexpression such as improved glucose tolerance^6^. Metabolic function is closely linked to oxidative stress. The mitochondria is the major cellular source of free radical production and as a consequence is highly susceptible to oxidative damage^17^. A number of studies have reported that enhancement of oxidative stress resistance in mice can increase metabolism and protect against high-fat diet-induced metabolic dysfunction, for example, catalase artificially targeted to the mitochondria and PRX3 have both been reported to increase metabolism and protect against high-fat diet-induced insulin resistance and glucose intolerance respectively. However, the mechanism by which these improvements were produced is unclear^5,6^.

Furthermore, our findings suggest TrxR2 overexpression could alleviate age-related declines in metabolic function. In previous work, we found TrxR2 function to be elevated in species which are under selection to be longer lived and that artificial overexpression of TrxR2 extended lifespan in fruit flies. While we did not see any improvement from overexpression of the cytosolic variant TrxR1. In addition, we found TrxR2 overexpression to decrease the age-related decline in muscle function and oxygen consumption in *Drosophila* suggesting prevention of age-related metabolic declines^14^.

Here, we demonstrated that TrxR2 overexpression in mice enhances whole-body metabolism. We found this enhancement to improve glucose tolerance by increasing the rate of blood glucose clearance, increasing tolerance to a high-fat diet, and decreasing the degree of high-fat diet-induced liver steatosis. We also showed this to occur in tandem with an increase in whole-body O_2_ consumption, CO_2_ production, and energy expenditure during the daytime suggesting an increase in basal metabolic rate. The observation that TrxR2-Tg high-fat diet-fed mice did not show any weight improvements was unexpected, however, this could be easily explained by a reactive oxygen species (ROS) overload caused by the diet. It has been reported that a long period of exposure to a high-fat diet induces a large production of ROS^18^ and the ability of overexpressed TrxR2 to clear them in tissues such as white fat might be affected in this condition.

We were interested to find the improvements in whole-body metabolism from TrxR2 overexpression were driven by changes in multiple mitochondrial metabolic processes. We observed all respiratory parameters to be enhanced in TrxR2-Tg isolated liver mitochondria. We found several components of the electron transport chain to show elevated activity including Complex I and V by both respiratory and direct activity measures. Complex IV respiration showed significantly enhanced function through seahorse assays with a positive trend in direct kinetic activity assays. We additionally found significant improvements in the output of several enzymes of the TCA cycle. This demonstrates a synergistic role for TrxR2 protein to increase mitochondrial function at different levels. Previous findings in plants have shown thioredoxin to be capable of modulating multiple components of the TCA cycle including malate dehydrogenase (MDH2) and isocitrate dehydrogenase (IDH2)^19^. Indeed, we found the functionality of both of these components to be elevated with TrxR2 overexpression. In addition, previous proteomics reports have pointed to specific components of the electron transport chain and TCA cycle to be targets of the cytoplasmic thioredoxin-1. Several subunits of Complex I, and the ε chain of ATP Synthase contain cysteines with thiol groups that are reduced by thioredoxin-1^16^ that seem to be impacting protein functionality and also be targets of thioredoxin-2.

All these results suggest metabolic improvements from augmentation of the thioredoxin system not to be through general declines in oxidative stress but to be through a targeted role of Thioredoxin-2 in reducing oxidized enzymatic components of both the electron transport chain and the TCA cycle. These results underscore the importance of the redox state as a key regulator of metabolic output and the sensitivity of its components to oxidative stress. These findings demonstrate the role of the mitochondrial thioredoxin system as a regulator of electron transport chain function, mitochondrial activity, and metabolism. Based on these findings and our rodent studies we can conclude that TrxR2 overexpression increases overall metabolism at least in part through increased electron transport chain and TCA function leading to increased glucose tolerance and metabolism.

## Methods

### Mouse model

We generated a CAG-Lp-STOP-Lp-TrxR2 construct which was microinjected by the University of Michigan transgenic core. Male mice containing the transgene in a CB6F1/C57Bl/6 J background were crossed with a homozygous CRE EIIa female germline overexpressor mouse (Cat# 003724, Jackson Labs). The EIIa-Cre mice carry a Cre transgene under the control of a zygotically expressed (EIIa-Cre) promoter that activates the expression of Cre recombinase in the early mouse embryos^20^. This resulted in a first-generation with 50% transgene expression. We then crossed positive mice with wild-type C57BL/6J for two more generations in order to avoid mosaicism in the following generations. From the second generation, we used both transgenic and non-transgenic littermates for our experiments. We genotyped transgenic positive mice with a forward primer (gtcaagctgcacatctccaa) and a reverse primer (gcgatgcaatttcctcattt) by PCR. We put a group of male and female mice under a normal diet and or high-fat diet (60% of fat, Research Diets cat#D12492) for 4 months. Animals were housed in ventilated cage racks with up to five animals per cage under 12 h light/dark cycles at 24 °C. Animals received daily monitoring by Laboratory of Animal Research (LAR) staff and were transferred to new cages weekly. Animal care and procedures were undertaken under protocol 2017004AR which was approved by UT Health San Antonio IACUC.

### Cell culture

Mouse Embryonic Fibroblasts were derived at E14 from CAG-Lp-STOP-Lp-TrxR2 male mice crossed with EIIa-CRE female mice. The cells were maintained in DMEM at 3% O_2_ and 5% CO_2_. ambient concentrations and 37 °C.

### Immunofluorescence

Cells were seeded on a coverslip then, after 24 h, incubated with 200 µM Mitotracker deep red (Thermofisher, M22426) for 15 min, fixed with 4% paraformaldehyde, washed with PBS, blocked with normal goat serum, and permeabilized with 0.3% Triton. Cells were incubated with the TrxR2 antibody (Lifespanbio, LS‑C118624, 1/200), washed, and incubated with goat anti-rabbit Alexa Fluor 488 (Thermofisher A-11008, 1/200), washed again and mounted with vectashield.

### Cell survival assays

Cells were seeded in 96-well plates using the manufacturer’s instructions WST-1 (Sigma 5015944001). Cells were treated with 100 and 500 µM H_2_O_2_ and *tert*-butyl hydroperoxide for 1 h, then cells were washed and incubated with WST-1 reagent for 30 min.

### Mitochondrial isolation

Mitochondria were isolated following a modification of the method by Rasmussen et. al.^21^ using a custom homogenizer. Freshly isolated mouse liver was immediately submerged in ice-cold B1 buffer (10 mM EGTA, 0.1 uM free calcium, 20 mM imidazole, 20 mM taurine, 50 mM K-MES, 6.56 mM MgCl_2_, 5.77 mM ATP, 15 mM phosphocreatine) containing protease inhibitor. The tissue was weighed and carefully cut into 1 mm cubes with a 9 mm razor blade while submerged in B1 buffer in a petri dish on ice. The sample was homogenized with a Craftsman drill press at 990 rpm using a customized Wheaton mortar and pestle, designed to standardize optimal and consistent yields and functionality in mitochondrial preparations^22^. The samples were maintained between 0–1 °C in a custom-built clear water-jacketed chamber. Homogenization included 12 slow passes of the pestle at 30 s for each downstroke, 30 s stopping of rotation once to the bottom, and 30 s on the upward pass. Homogenates were centrifuged at 600 × *g*, 10 min, at 4 °C. The supernatants were transferred to another ice-cold centrifuge tube and centrifuged at 10,000 × *g*, 10 min, at 4 °C. The pellet was then rinsed with 50 µL ice-cold isolation buffer and the supernatant removed. The final pellet was gently resuspended in 100 µL isolation buffer and protein concentration (mg/mL) was determined by Lowry quantification.

### Seahorse assays

The Seahorse XF96 sensor cartridge (Agilent Technologies, Seahorse Bioscience) was hydrated in sterile water in a non-CO_2_ 37 °C incubator overnight and then in a pre-warmed calibrant in a non-CO_2_ 37 °C incubator 1 h before the assay run. About 20 µg of mitochondria were resuspended in MiR03 buffer (20 mM sucrose, 10 mM KH_2_PO_4_, 3 mM MgCl_2_-6H_2_O, 20 mM HEPES, 0.5 mM EGTA, 0.1% (w/v) fatty acid-free BSA, 20 mM taurine) with 10 mM pyruvate and 2 mM malate was used to dilute effectors for both the coupling assay (CA) and the electron flow assay (EFA), with 150 µM 2,4-dinitrophenol (DNP) added to the EFA buffer. All effectors were made to 10× desired concentrations and loaded into the appropriate ports of the sensor cartridge at 20, 22, 25, and 27 µL in ports A, B, C, D respectively for the CA assay (port A: 40 mM ADP, port B: 25 µg/mL oligomycin, port C: 1500 µM DNP, port D: 40 µM antimycin A) and the EFA assay (port A: 20 µM rotenone, port B: 100 mM succinate, port C: 40 µM antimycin A, port D: 100 mM ascorbate and 1 mM TMPD). About 20 µg mitos were seeded in 50 µL CA or EFA buffer in the designated wells of the XF96 tissue culture microplate and then centrifuged at 2000 × *g* for 20 min. About 130 µL of CA or EFA buffer was gently added to each well and the plate was loaded into the Seahorse XFe96 Analyzer (Agilent Technologies, Seahorse Bioscience). The Seahorse protocol was run as follows: calibrate (15 min), equilibrate (15 min), 3× basal reads (Mix: 3 min, Wait: 0 min, Measure: 3 min), then a port injection (A, B, C, D) each followed by 3× reads. Oxygen consumption rate (OCR) and extracellular acidification rate (ECAR) was obtained at baseline and in response to each effector injection.

### Mitochondrial activity assays

All assays were performed using 30 µg of isolated mitochondria. Complex I activity was immediately measured on a DU800 spectrophotometer using 2,6-dichloroindophenol (DCIP) as the terminal electron acceptor at 600 nm with the oxidation of NADH reducing artificial substrates Coenzyme Q10 that then reduces DCIP. The reduction of DCIP is mostly dependent on complex I activity and has a very high rotenone-sensitive activity (1). Complex II activity will be analyzed as the reduction of dichloroindophenol at 600 nm with succinate as the substrate, and complex II/III will be measured as the reduction of cytochrome *c* at 550 nm also with succinate as the substrate (2). Complex IV activity was measured by the oxidation of cytochrome c at 550 nm (3). Data were represented as the pseudo-first-order rate constant (k) divided by protein concentration. ATP synthase activity, measured in the direction of ATP hydrolysis (ATPase activity), was assayed by the continuous spectrophotometric monitoring of the oxidation of NADH (e340 = 6180 M^−1^ cm^−1^) in an enzyme-linked ATP regenerating assay using ATP, phosphoenolpyruvate, pyruvate kinase, and lactate dehydrogenase to determine the ATPase activity (NADH loss) in nmol/min/mg protein (4, 5). Citrate synthase was measured using the coupled reaction with oxaloacetate, acetyl-CoA, and 5,5-dithiobis-(2,4-nitrobenzoic acid) (6).

### Mitochondrial membrane potential

Mitochondrial membrane potential was monitored with a Zeiss LSM740 confocal microscope, cells were seeded in glass-bottom dishes and incubated in 10 nM TMRM (non-quenching mode) for 15 min in Seahorse XF DMEM medium (Agilent part 103575) containing 10 mM glucose, 1 mM sodium pyruvate, and 2 mM l-glutamine without phenol red. For H_2_O_2_ treatment cells were incubated with 100 μM H_2_O_2_ 30 min prior to the TMRM incubation. Cells were imaged immediately after TMRM incubation.

### Malate dehydrogenase (MDH) and succinate dehydrogenase (SDH) activities

These measures were performed in mouse liver mitochondria that were isolated by differential centrifugation. Livers were homogenized in MSHE buffer (70 mM sucrose, 210 mM mannitol, 5 mM HEPES, 1 mM EGTA pH 7.2, and spun at 800 × *g* for 10 min. The supernatants were then centrifuged at 8000 × *g* for 10 min in order to obtain the mitochondrial pellet. Mitochondria were then washed and resuspended in MSHE for protein contraction. About 1 μg of mitochondria were used to measure MDH and SDH activities following the manufacturer’s instructions by Abcam (ab183305 and ab102528 kits) in 96-well colorimetric assays

### Western blots

SDS-PAGE was performed using the Biorad precast gels and transferred into PVDF membranes. Blots were probed with TrxR2 primary (Millipore, SAB450013, 1/5000), PGC-1α (Abcam, ab106814, 1/5000), VDAC (Abcam, ab14734, 1/5000), Prx3 (Abcam, ab73349, 1/1000), Prx_OX_ (Abcam, ab16830, 1/1000) and secondary HRP-linked anti-rabbit (Cell Signaling, 7074, 1/10,000), HRP-linked anti-mouse (Cell Signaling, 7076, 1/10,000), IRDye 680LT anti-rabbit (Licor, 926-68021, 1/5000) or IRDye 800CW anti-mouse (Licor, 926-32210, 1/5000).

### GTT and ITT assays

Glucose and Insulin Tolerance Tests were performed using an Aimstrip plus glucometer. A bolus of 1.5 g/kg bolus of glucose or a 0.8 U/kg bolus of insulin were injected intraperitoneally into mice that were starved overnight. Then glucose was monitored for a total period of 2 h. Procedures were approved under IACUC 20170040AR.

### qMRI and indirect calorimetry and food intake

The whole-body composition was obtained with EchoMRI-100H and 130 (EchoMRI). Measures of total body fat, lean mass, and free water as grams of body weight were obtained in individual awake animals (anesthesia was not required). We ensured that mice were immobile and located at the bottom of the measurement tube. For calorimetry Mice were individualized in cages and left to acclimate in the room for two days. We used the MARS indirect calorimetry “pull mode” system (Sable Systems) to determine whole-body metabolic parameters such as O_2_ consumption, CO_2_ production, and heat production for a period of 48 h (three light and two dark phases). Data from the third hour is examined to detect potential equipment-related problems so that animals can be quickly retested.

Food Intake was recorded by weighing the food at the beginning of the week and then weighing the daily changes. Then the final sum of the daily changes was added for 7 days in order to plot the differences.

### Statistics and reproducibility

All statistical tests applied were *t*-Student’s tests unless otherwise specified. The number of samples or mice utilized are specified for each figure panel. Normally at least three biological samples were used with three different technical replicates. For animal physiological measures, seven to nine animals were used. Significance was considered for *p* values ≤0.05.

### Reporting summary

Further information on research design is available in the Nature Research Reporting Summary linked to this article.

## Supplementary information

Supplementary Information

Reporting Summary

## Data Availability

The datasets generated during and/or analyzed during the current study are available in the Dryad repository, doi:10.5061/dryad.fttdz08vk, Uncropped gels are available in Supplementary Fig. 6.
