# Supplementary material for: Mitochondrial TrxR2 regulates metabolism and protects from metabolic disease through enhanced TCA and ETC function
Source: Commun Biol. 2022 May 16;5:467. doi: 10.1038/s42003-022-03405-w (PMC9110405; doi:10.1038/s42003-022-03405-w)
Supplement: Supplementary file 1 — Supplementary Information [file 42003_2022_3405_MOESM1_ESM.pdf]

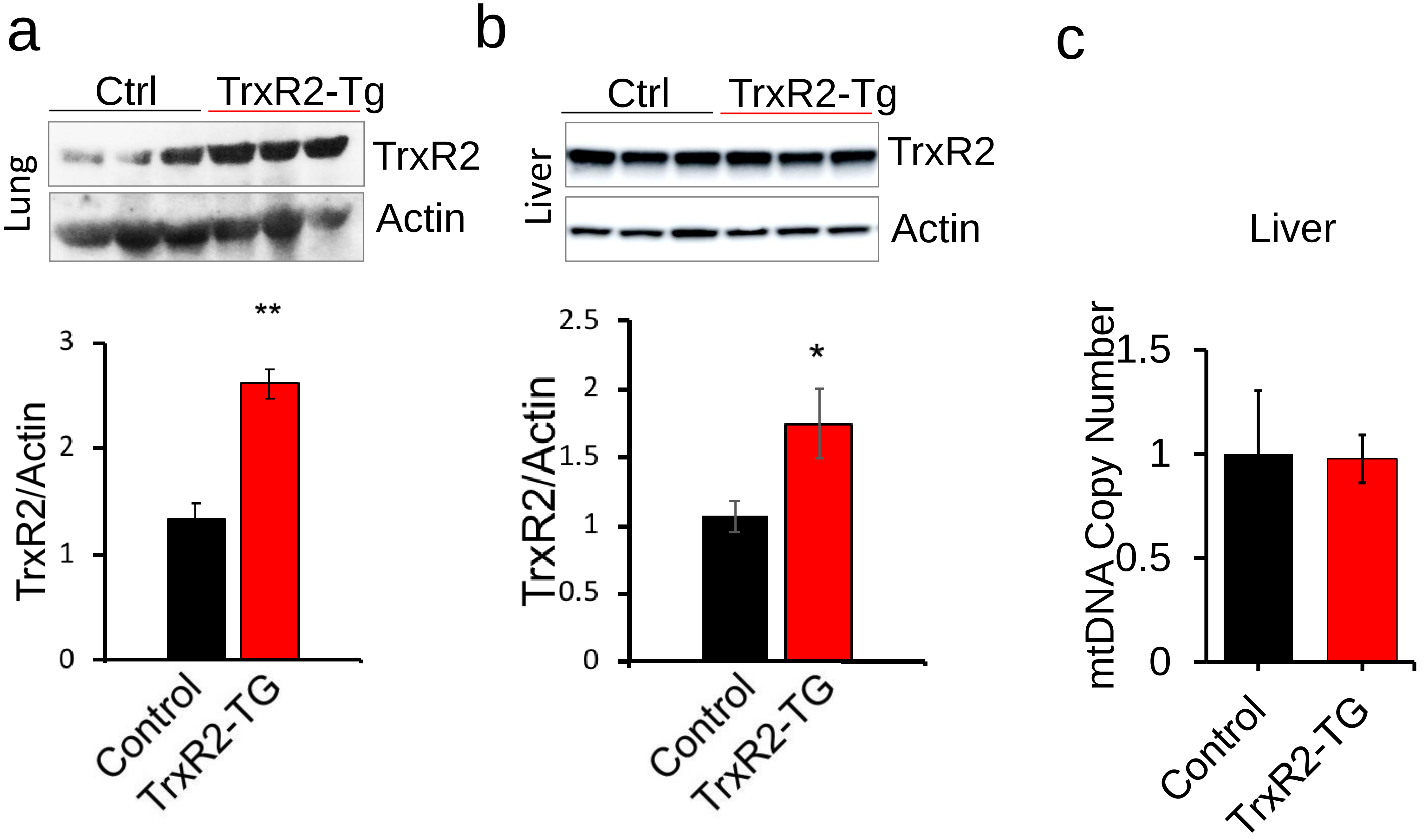

Supplemental Fig. 1: Supplemental characterization of TrxR2-Tg mouse. (a-b) TrxR2 is elevated in TrxR2-Tg mice. Immunoblots and quantifications of TrxR2 in lung and live tissues from 10-month-old TrxR2-Tg mice and littermate controls (n=3). (c) TrxR2 overexpression does not alter mitochondrial abundance. Relative PCR ratio of ND1 (mtDNA maker) vs HK2 (nDNA marker) in liver tissue from 10-month-old TrxR2-Tg mice and littermate controls (n=3). Values are Mean ± SEM. * $p < 0.05$, ** $p < 0.01$.

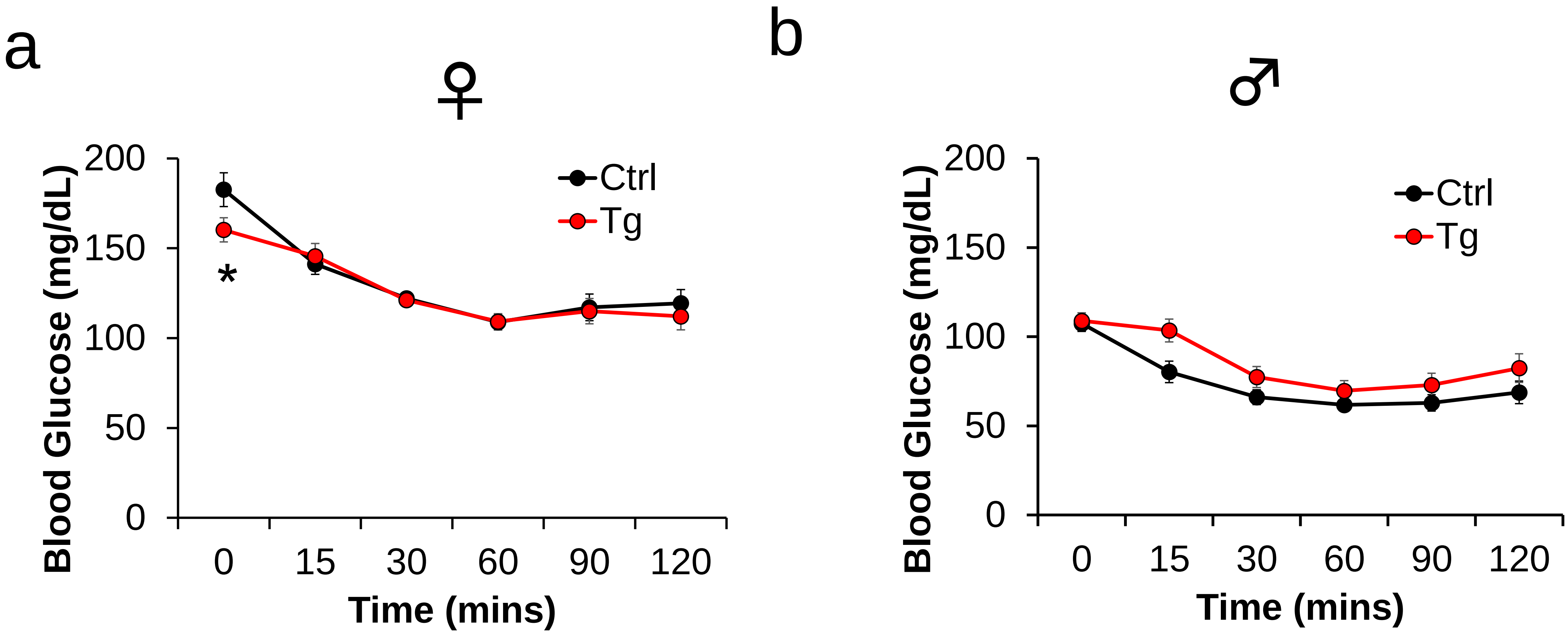

Supplemental Fig. 2: Insulin sensitivity in high-fat diet fed TrxR2-Tg mice compared to controls. Insulin sensitivity test in High-fat diet fed 8 months old TrxR2-Tg and littermate controls. Separate tests were done for female (a) and male (b) groups (n = 8-9). Values are Mean ± SEM. * $p < 0.05$, ** $p < 0.01$.

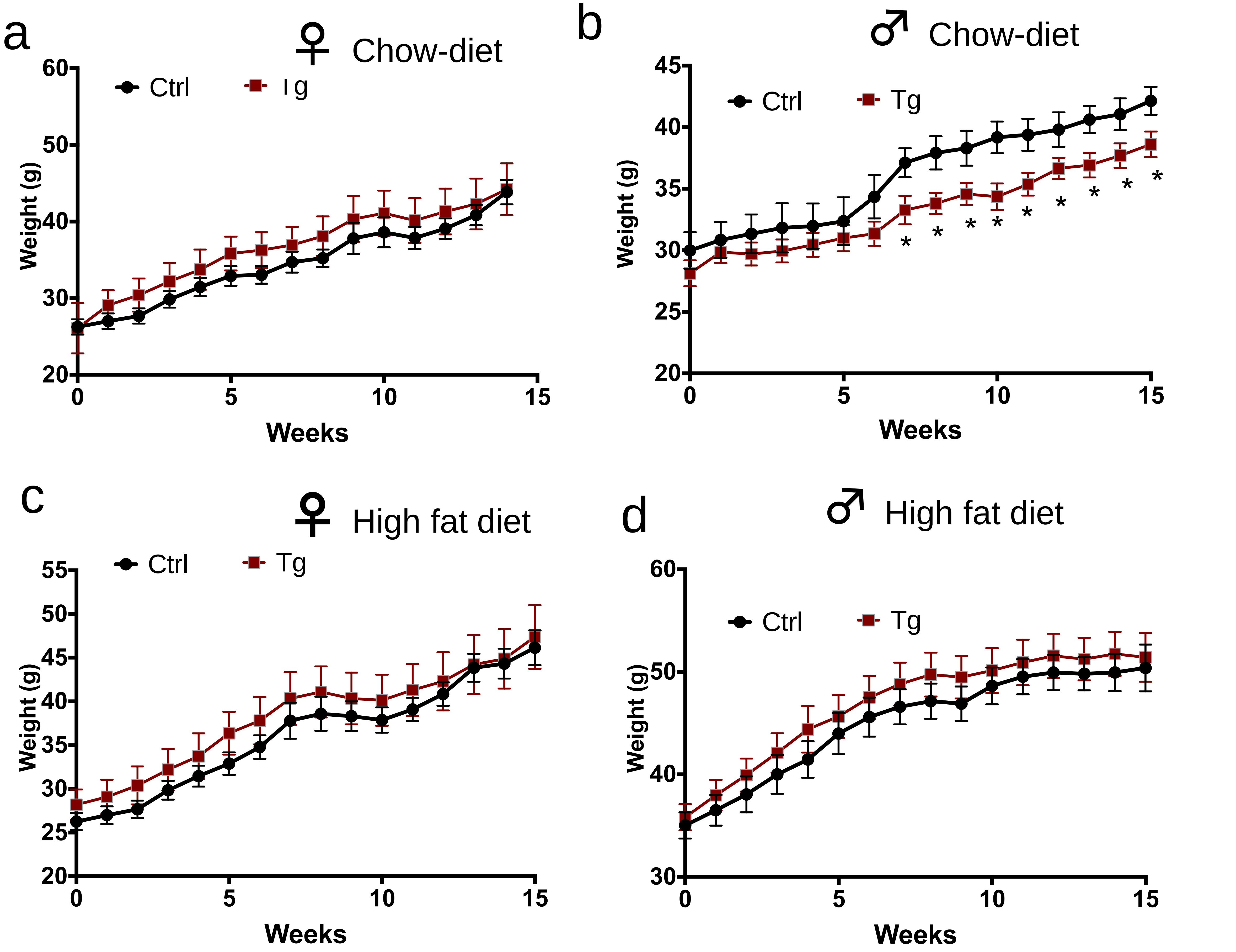

Supplemental Fig. 3: Mouse weight. Weight was monitored weekly from 3 Mo (0 time point) when high fat diet treatment was started. Weight unchanged in chow fed females (a), weight decreased in chow fed males (b), weight unchanged in high fat diet fed females (c) and males (d). n = 7-9. Values are Mean ± SEM. * $p < 0.05$.

Supplemental Fig. 4

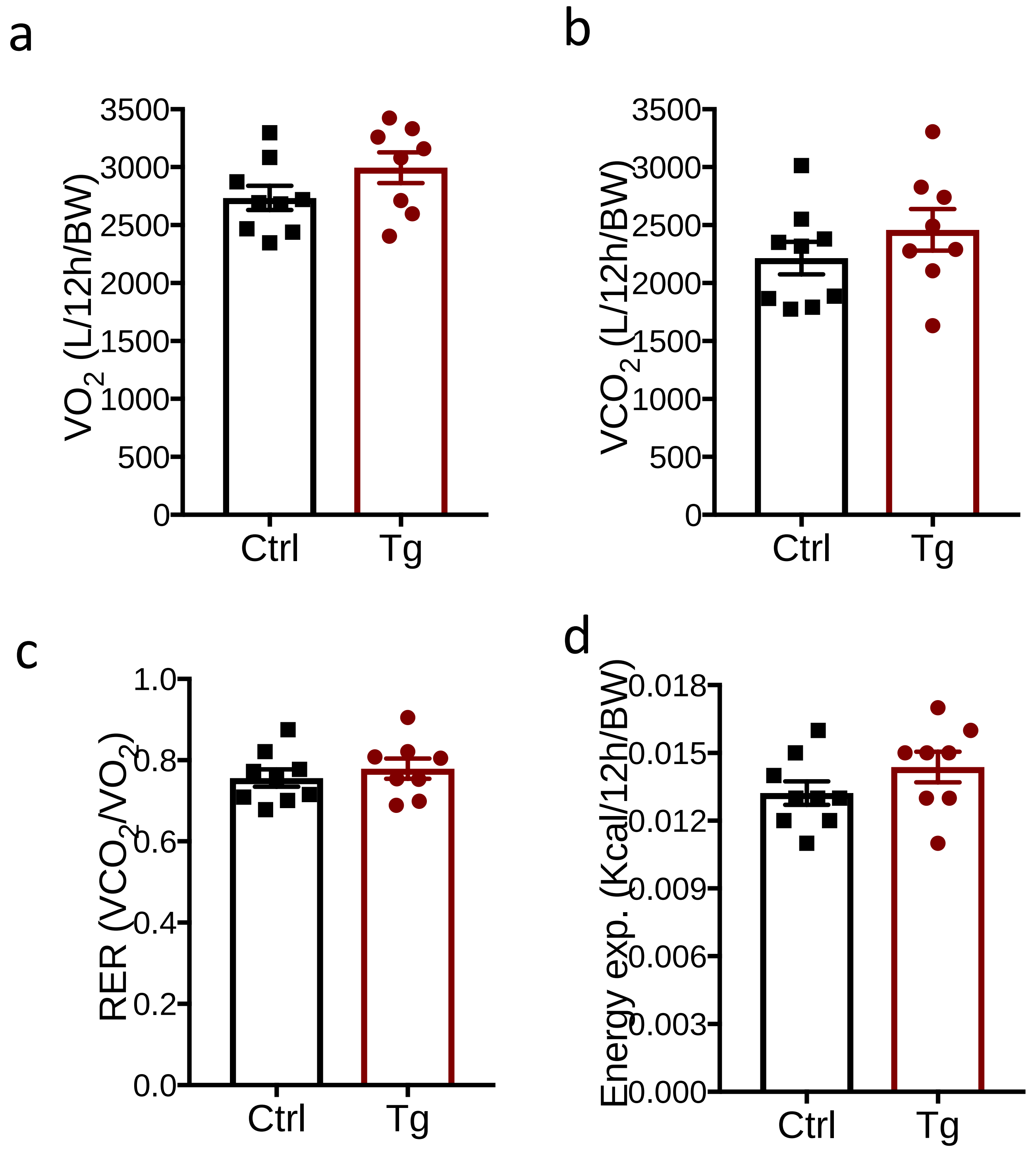

Supplemental Fig. 4: TrxR2 overexpression did not alter dark cycle whole-body metabolism in normal diet fed male mice. Indirect calorimetry was performed in normal fed male mice. Parameters for each mouse were obtained and normalized by body weight. (**a**) Night time O_2 consumption (VO_2) **(b)**, CO_2 production (VCO_2). **(c)** RER (CO_2/O_2) (**d**) and energy expenditure (Heat). All experiments utilized 8 months old normal fed male mice, n = 8-9. Values are Mean ± SEM. * $p < 0.05$.

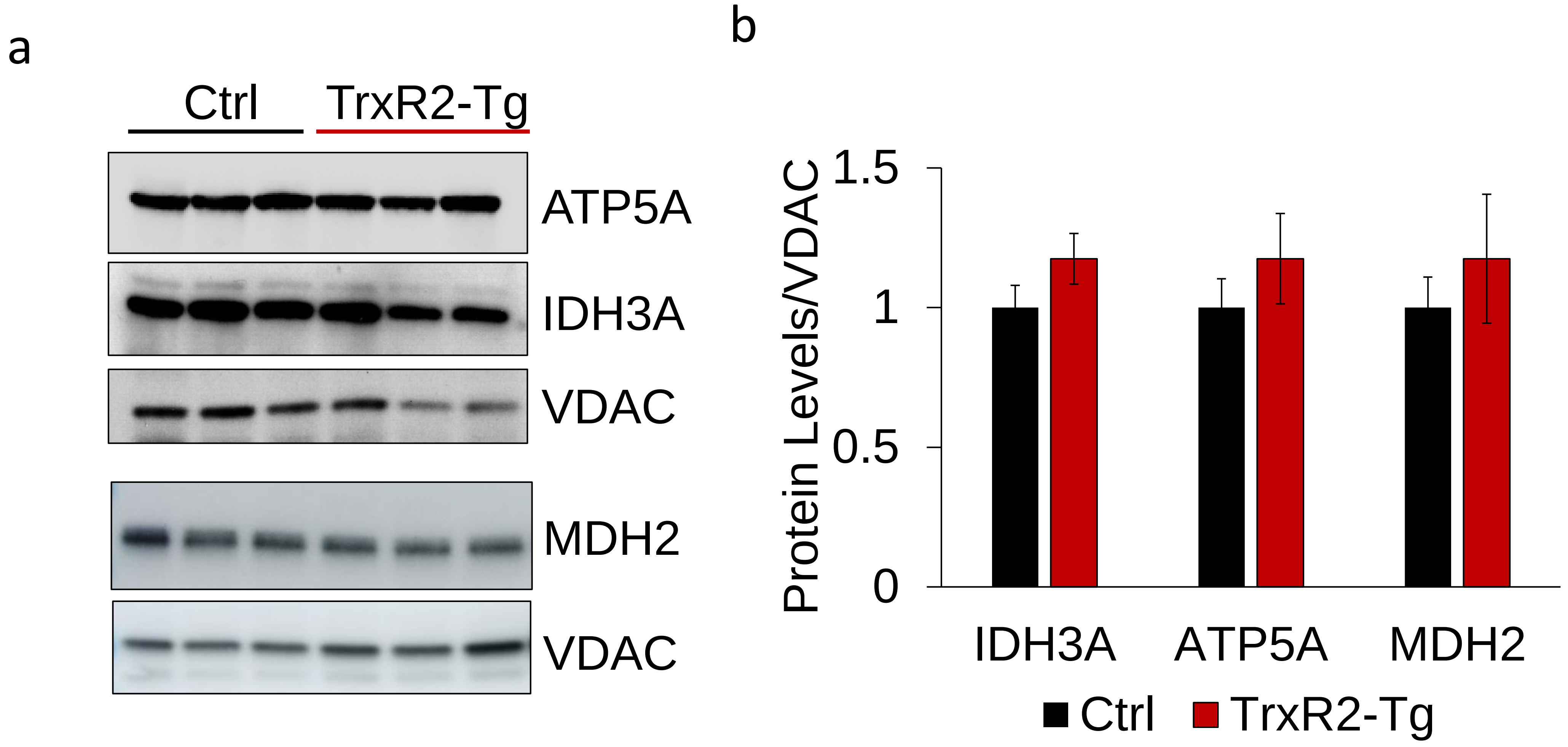

Supplemental Fig. 5. ATP5a, IDH3A and MDH2 protein levels unaltered. Immunoblot (a) and quantitation (b) of TCA enzymes ATP5A, MDH2 and IDH2. Values normalized to VDAC (n = 3). Values are Mean ± SEM. * $p < 0.05$, ** $p < 0.01$

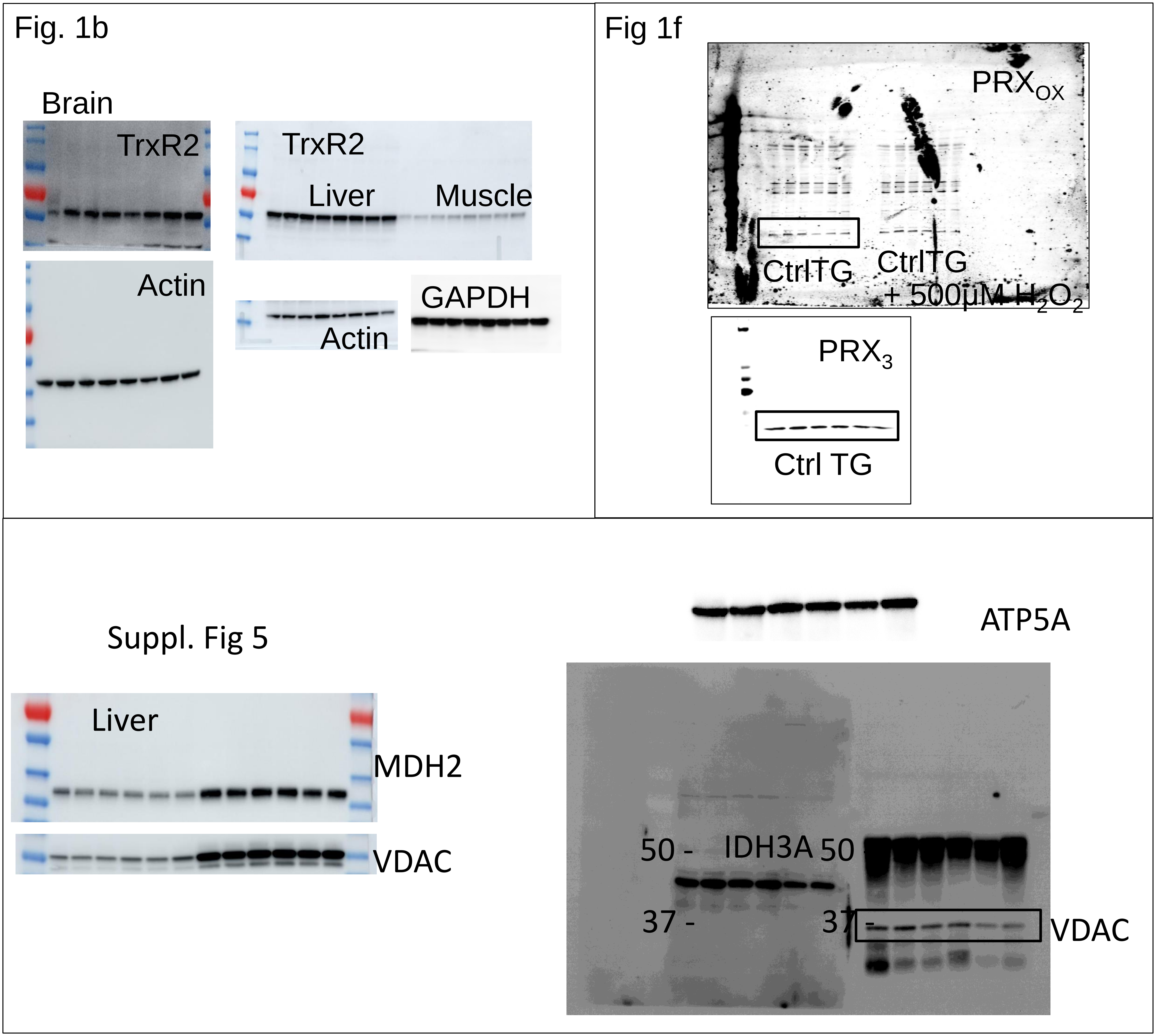

Supplemental Fig. 6: Uncropped gel images
