# Supplementary material for: Mitochondrial TrxR2 regulates metabolism and protects from metabolic disease through enhanced TCA and ETC function
Source: Commun Biol. 2022 May 16;5:467. doi: 10.1038/s42003-022-03405-w (PMC9110405; doi:10.1038/s42003-022-03405-w)
Supplement: Supplementary file 2 — Reporting Summary [file 42003_2022_3405_MOESM2_ESM.pdf]

nature portfolio

Corresponding author(s): Andrew M. Pickering

Last updated by author(s): Jan 12, 2022

Reporting Summary

Nature Portfolio wishes to improve the reproducibility of the work that we publish. This form provides structure for consistency and transparency in reporting. For further information on Nature Portfolio policies, see our Editorial Policies and the Editorial Policy Checklist.

Statistics

For all statistical analyses, confirm that the following items are present in the figure legend, table legend, main text, or Methods section.

n/a	Confirmed	
☐	☒	The exact sample size (*n*) for each experimental group/condition, given as a discrete number and unit of measurement
☐	☒	A statement on whether measurements were taken from distinct samples or whether the same sample was measured repeatedly
☐	☒	The statistical test(s) used AND whether they are one- or two-sided<br>*Only common tests should be described solely by name; describe more complex techniques in the Methods section.*
☐	☒	A description of all covariates tested
☐	☒	A description of any assumptions or corrections, such as tests of normality and adjustment for multiple comparisons
☐	☒	A full description of the statistical parameters including central tendency (e.g. means) or other basic estimates (e.g. regression coefficient) AND variation (e.g. standard deviation) or associated estimates of uncertainty (e.g. confidence intervals)
☐	☒	For null hypothesis testing, the test statistic (e.g. *F*, *t*, *r*) with confidence intervals, effect sizes, degrees of freedom and *P* value noted<br>*Give P values as exact values whenever suitable.*
☒	☐	For Bayesian analysis, information on the choice of priors and Markov chain Monte Carlo settings
☒	☐	For hierarchical and complex designs, identification of the appropriate level for tests and full reporting of outcomes
☒	☐	Estimates of effect sizes (e.g. Cohen's *d*, Pearson's *r*), indicating how they were calculated

Our web collection on statistics for biologists contains articles on many of the points above.

Software and code

Policy information about availability of computer code

Data collection	imageJ, wave (seahorse),
Data analysis	SigmaStat, microsoft office, imageJ

For manuscripts utilizing custom algorithms or software that are central to the research but not yet described in published literature, software must be made available to editors and reviewers. We strongly encourage code deposition in a community repository (e.g. GitHub). See the Nature Portfolio guidelines for submitting code & software for further information.

Data

Policy information about availability of data

All manuscripts must include a data availability statement. This statement should provide the following information, where applicable:

- Accession codes, unique identifiers, or web links for publicly available datasets
- A description of any restrictions on data availability
- For clinical datasets or third party data, please ensure that the statement adheres to our policy

The datasets generated during and/or analysed during the current study are available from the corresponding author on reasonable request.

Field-specific reporting

Please select the one below that is the best fit for your research. If you are not sure, read the appropriate sections before making your selection.

☒ Life sciences ☐ Behavioural & social sciences ☐ Ecological, evolutionary & environmental sciences

For a reference copy of the document with all sections, see nature.com/documents/nr-reporting-summary-flat.pdf

Life sciences study design

All studies must disclose on these points even when the disclosure is negative.

Sample size	Animal studies utilized an N of 7-10, based on prior studies we expected N = 6 to be sufficiently powered for our endpoints.
Data exclusions	Extreme outliers which exceeded 2 standard deviations were excluded from the dataset
Replication	All replications attempts were successful
Randomization	Most tests were based on animal genotype which cannot be randomized. Assignment to high-fat diet vs chow diet was based on cage. Cage assignment was based on ensuring as equal as possible distribution of control and transgenic animals between groups and ensuring an equal age distribution.
Blinding	For GTT and ITT tests the treatment and recording investigator was blinded. For Seahorse assays the investigator was blinded.

Reporting for specific materials, systems and methods

We require information from authors about some types of materials, experimental systems and methods used in many studies. Here, indicate whether each material, system or method listed is relevant to your study. If you are not sure if a list item applies to your research, read the appropriate section before selecting a response.

Materials & experimental systems

n/a	Involved in the study	
☐	☒	Antibodies
☐	☒	Eukaryotic cell lines
☒	☐	Palaeontology and archaeology
☐	☒	Animals and other organisms
☒	☐	Human research participants
☒	☐	Clinical data
☒	☐	Dual use research of concern

Methods

n/a	Involved in the study	
☒	☐	ChIP-seq
☒	☐	Flow cytometry
☒	☐	MRI-based neuroimaging

Antibodies

Antibodies used	TrxR2 antibody (Lifespanbio, LS-C118624), anti-rabbit Alexa Fluor 488 (Thermofisher A-11008), MDH (abcam ab183305), SDH (abcam ab102528), TrxR2 (Millipore Sigma SAB450013), PGC-1α (abcam ab106814) and VDAC (Abcam ab14734).
Validation	Antibodies validated by manufacturer with in-house molecular weight confirmation for immunoblots.

Eukaryotic cell lines

Policy information about cell lines

Cell line source(s)	primary MEF cells derived in-house
Authentication	Cells were derived in-house and used within P3, authentication unnecessary.
Mycoplasma contamination	Cells were not tested
Commonly misidentified lines (See ICLAC register)	N/A

Animals and other organisms

Policy information about studies involving animals; ARRIVE guidelines recommended for reporting animal research

Laboratory animals	CAG-TXNRD2, CAG-LoxP-STOP-LoxP-TXNRD2, EIIA-CRE, C57BL/6J, CB6F1
Wild animals	NA
Field-collected samples	NA
Ethics oversight	Animal care and procedures were done undertaken under protocol 2017004AR which was approved by UT Health San Antonio IACUC.

Note that full information on the approval of the study protocol must also be provided in the manuscript.
